# Filamin C, a dysregulated protein in cancer revealed by label-free quantitative proteomic analyses of human gastric cancer cells

**DOI:** 10.18632/oncotarget.2645

**Published:** 2014-11-28

**Authors:** Jie Qiao, Shu-Jian Cui, Lei-Lei Xu, Si-Jie Chen, Jun Yao, Ying-Hua Jiang, Gang Peng, Cai-Yun Fang, Peng-Yuan Yang, Feng Liu

**Affiliations:** ^1^ Department of Medical Systems Biology of School of Basic Medical Sciences and Institutes of Biomedical Sciences, Fudan University, Shanghai 200032, China; ^2^ Department of Chemistry, Fudan University, Shanghai 200433, China; ^3^ College of Bioscience and Biotechnology, Key Laboratory of Crop Genetics and Physiology of Jiangsu Province, Yangzhou University, Yangzhou 225009, China; ^4^ Institutes of Brain Science, Fudan University, Shanghai 200032, China

**Keywords:** Gastric cancer, Proteomics, Filamin C, Metastasis, Tumor suppressor

## Abstract

Gastric cancer (GC) is the fourth and fifth most common cancer in men and women, respectively. We identified 2,750 proteins at false discovery rates of 1.3% (protein) and 0.03% (spectrum) by comparing the proteomic profiles of three GC and a normal gastric cell lines. Nine proteins were significantly dysregulated in all three GC cell lines, including filamin C, a muscle-specific filamin and a large actin-cross-linking protein. Downregulation of filamin C in GC cell lines and tissues were verified using quantitative PCR and immunohistochemistry. Data-mining using public microarray datasets shown that filamin C was significantly reduced in many human primary and metastasis cancers. Transient expression or silencing of filamin C affected the proliferation and colony formation of cancer cells. Silencing of endogenous filamin C enhanced cancer cell migration and invasion, whereas ectopic expression of filamin C had opposing effects. Silencing of filamin C increased the expression of matrix metallopeptidase 2 and improved the metastasis of prostate cancer in a zebrafish model. High filamin C associated with better prognosis of prostate cancer, leukemia and breast cancer patients. These findings establish a functional role of filamin C in human cancers and these data will be valuable for further study of its mechanisms.

## INTRODUCTION

Gastric cancer (GC) is the fourth and fifth most common cancer in men and women, respectively, with an estimated incidence of 990,000 cases and 740,000 deaths in 2008 [1]. The mortality of GC is third for males and fifth for females. Wide regional differences are noted for both incidence and mortality, and GC rates have declined significantly in recent years due to many factors. Increased salt intake, red and processed meat intake, tobacco and alcohol consumptions have been considered as the environmental and behavioral risk factors for the development of GC [2, 3]. In addition, chronic infections caused by *Helicobacter pylori*, Epstein–Barr virus and human immunodeficiency virus may also increase the risk of GC [3].

It has been reported that mutations in a number of genes, such as the E-cadherin and alpha-E-catenin genes, contributed to GC pathogenesis [4, 5]. Recent exome sequencing have also identified that genes encoding the tumor protein p53 (TP53), phosphatidylinositol-4, 5-bisphosphate 3-kinase catalytic subunit alpha, AT rich interactive domain 1A, activin receptor type 2, ribosomal protein L22, and lectin, mannose-binding, 1 (LMAN1) were frequently mutated in GC patients [6–8]. In addition, over one hundred microRNAs, a family of small non-coding RNA molecules, have been shown to be involved in the carcinogenesis, metastasis, and drug resistance of GC [9]. Epigenetic mechanisms such as promoter or histone hypermethylation, which induces gene silencing, also contribute to the initiation and progression of GC [10]. Furthermore, genetic instabilities, such as microsatellite instability and chromosomal instability, are important genetic risk factors for early transformation of gastric cells [11]. Therefore, GC is a multifactorial disease in which numerous and complex genetic factors and mechanisms are involved.

Proteomics technologies have been widely used in GC studies. To profile proteins expressed at different stages or under different condition, two proteomic strategies are commonly applied, i.e. two-dimensional electrophoresis (2DE) [12] and liquid chromatograph-mass spectrometry (LC-MS) [13, 14]. To absolutely or relatively quantify the expression levels of proteins, both label-free and labeling methods have been developed and extensively used in GC studies, such as the multidimensional protein identification technology (MudPIT) [15], isotope coded affinity tag (ICAT) [16], 2DE-difference gel electrophoresis (DIGE) [17], isobaric tags for relative and absolute quantification (iTRAQ) [18], stable isotope labeling by amino acids in cell culture [19], and isobaric tandem mass tags [20]. Primary or metastatic tumor tissues, different GC cell lines, serum and biopsies extracted from GC patients through laser capture microdissection [13] have been subjected to proteomic analyses. For example, caldesmon has been identified as an important protein involved in the development of GC based on a proteomic study [21]. In addition, a number of potential prognostic markers, such as chemokine (C-C motif) ligand 18 and chemokine (C-X-C motif ) ligand 1 [22] have been identified on the basis of proteomic analyses. Therefore, proteomic analyses are powerful tools to identify key molecules involved in the development of GC as well as prognostic factors for GC patients.

In the present study, we compared the proteomic profiles of a normal gastric cell line and three GC cell lines based on label free LC-MS. We focused on the filamin C protein that was significantly downregulated in all six GC cell lines analyzed. Downregulation of filamin C was detected in primary and metastatic tumor tissues of GC and prostate cancers. Silencing of *filamin C* in GC or prostate cancer cell lines enhanced cell migration and invasion, whereas *filamin C* overexpression inhibited the migration and invasion of cancers cells. Our results suggest that filamin C is a tumor suppressor involved in the development of GC and prostate cancer.

## RESULTS

### Comprehensive proteomic analysis of GC cell expression by label-free LC-MS

**Figure 1:**
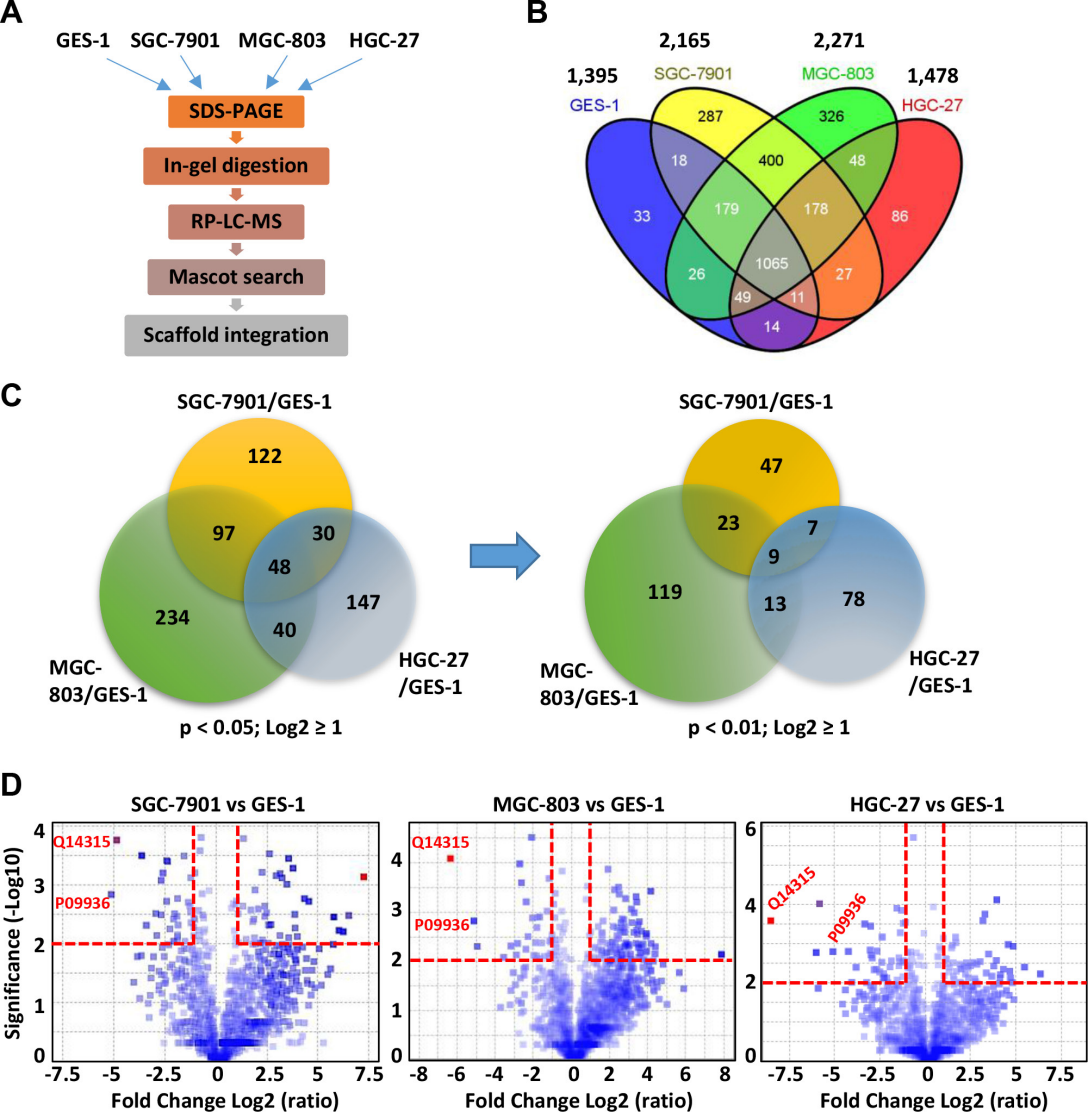
Proteomic analyses of a normal gastric cell line (GES-1) and three GC cell lines (SGC-7901, MGC-803, and HGC-27) **(A)** The general procedures of proteomic analyses. **(B)** The total number of proteins identified in each cell line and the overlaps among the four cell lines. **(C)** The Venn diagrams showing the overlaps of the differentially expressed proteins among the three GC cell lines. The differentially expressed proteins in each GC cell line were determined with a fold change log^2^ ratio ≥ 1 (i.e. fold change ≥ 2) and a *p* value of the Student's *t* test < 0.05 or < 0.01. To calculate the fold changes, the average expressions in the GC cell sets were divided by the average expressions in the GES-1 set and the ratios were Log^2^ transformed. **(D)** The volcano plots depict the differentially expressed proteins in the three GC cell lines. The *p* values of *t*-test (−log^10^ transformed) were displayed as a function of the log^2^ transformed fold changes. The red dash lines indicated the threshold of *p* < 0.01 and fold change ≥ 2. The accession numbers of filamin C (Q14315) and UCHL1 (P09936) were highlighted.

GES-1 is an immortalized stomach mucosal cell line established by SV40 virus infection of 9 month human fetal gastric epithelial cells [23], whereas SGC-7901, MGC-803, and HGC-27 represent the moderate-, low- and non-differentiated gastric cancer cell lines, respectively. The proteomic profiles of the four cell lines were analyzed using label free LC-MS with LTQ Obitrap in triplicates (Figure 1A, Supplementary Methods and Supplementary Table 2). A total of 2,787 proteins including 36 decoy hits were identified from 27,067 distinct peptides and 347,681 tandem spectra. The false discovery rates at protein and spectrum level reported by Scaffold were 1.3% and 0.03%, respectively. The information of identified peptides and proteins were shown in Supplementary Tables 3 and 4, respectively. Among the 2,750 proteins, 1,395, 2,165, 2,271, and 1,478 proteins were identified in GES-1, SGC-7901, MGC-803, and HGC-27, respectively, and 1,065 proteins were shared by the four cell lines (Figure 1B).

Compared with GES-1 cells, 297, 419, and 265 proteins were down-regulated or up-regulated by ≥ 2 folds (Log^2^ ≥ 1 or ≤ −1) with *p* value < 0.05 (−Log^10^ > 2) in SGC-7901, MGC-803, and HGC-27 cells, and 48 differentially expressed proteins were shared by the three GC cell lines (Figure 1C). Forty three proteins show consistent expression changes (up-regulation or down-regulation) throughout the three GC cell lines (Table 1). No significantly enriched pathways and function were identified for these 43 proteins based on Gene ontology (GO) enrichment analysis. When the *p* value was set as 0.01, the numbers of differentially expressed proteins in SGC-7901, MGC-803, and HGC-27 cells are reduced to 86, 164 and 107, respectively. Finally, 9 proteins that were significantly dysregulated (*p* < 0.01, > 2 folds) in all three GC cell lines were identified by comparing the expression profiles of three GC cell lines with that of GES-1 cells (Figure 1C). The calculation results were shown in Supplementary Table 4. The volcano plot of the −log^10^ of the *p* value of T-test as a function of log^2^ fold change for each protein was shown in Figure 1D. All the 9 proteins were downregulated in three GC cell lines compared to the GES-1 cell line, including glycogen phosphorylase (PYGL), ubiquitin carboxyl-terminal hydrolase isozyme L1 (UCHL1), ephrin type-A receptor 2 (EPHA2), transgelin, filamin C, UDP - N-acetylhexosamine pyrophosphorylase (UAP1), HEAT repeat-containing protein 2 (HEATR2), lysophospholipid acyltransferase 7 (MBOAT7), and nucleolar protein 16 (NOP16). Among them, the quantitative values of PYGL, UCHL1, transgelin, and filamin C in GES-1 cells were over 40, while the others 5 proteins have relatively low quantitative values (Supplementary Table 4). It should be noted that higher quantitative values of proteins are associated with stronger reliability of the dysregulation of proteins in GC cell lines. The proteins filamin C (UniProt accession Q14315) and UCHL1 (UniProt accession P09936) represented the obvious differential proteins as these proteins were only detected in GES-1. Filamin C exhibited the highest quantitative value (> 400) in GES-1 cell, but extremely low quantitative values in all three GC cell lines (Figure 1D). GO analysis revealed that filamin C was associated with cell junction assembly and muscle fiber development (Supplementary Table 4). Therefore, the present study focused on the role of protein filamin C in the development of GC and other human cancers.

**Table 1:** Differential proteins between GES-1 and three GC cell lines revealed by proteomic analysis

Name	Accession	MW (kDa)	Cov.^a^	FC. (log2)/*p* value^b^		
				SGC7901^c^	MGC803	HGC27
Filamin-C (FLNC)	Q14315	291	44%	−5.02/0	−6.52/0	−8.71/0
Glycogen phosphorylase, liver form (PYGL)	P06737	97	32%	−2.75/0	−1.18/0	−2.56/0
Transgelin (TAGLN)	Q01995	23	64%	−3.1/0	−5.11/0	−3.17/0
Ubiquitin carboxyl-terminal hydrolase isozyme L1 (UCHL1)	P09936	25	64%	−5.29/0	−5.29/0	−5.29/0
UDP-N-acetylhexosamine pyrophosphorylase (UAP1)	Q16222	59	29%	−2.85/0	−2.06/0	−3.52/0
Lysophospholipid acyltransferase 7 (MBOAT7)	Q96N66	53	15%	−2.01/0	−3.73/0.01	−2.83/0
Nucleolar protein 16 (NOP16)	Q9Y3C1	21	40%	−2.58/0	−1.22/0.01	−3.42/0.01
HEAT repeat-containing protein 2 (HEATR2)	Q86Y56	94	8%	−2.57/0	−3/0	−1.04/0.01
Ephrin type-A receptor 2 (EPHA2)	P29317	108	7%	−2.52/0	−1.92/0	−2.88/0
26S proteasome non-ATPase regulatory subunit 2 (PSMD2)	Q13200	100	36%	−2.04/0.01	−1.22/0.01	−1.73/0.01
Eukaryotic translation initiation factor 6 (EIF6)	P56537	27	64%	−1.23/0.02	−1.07/0.02	−1.91/0.01
Cation-independent mannose-6-phosphate receptor (IGF2R)	P11717	274	11%	−3.79/0	−2.45/0	−2.47/0.01
Protein phosphatase methylesterase 1 (PPME1)	Q9Y570	42	42%	−1.6/0.01	−2.56/0	−4.59/0.01
Copine-3 (CPNE3)	O75131	60	25%	−1.44/0.02	−1.83/0	−3.2/0
Tripeptidyl-peptidase 2 (TPP2)	P29144	138	10%	−2.4/0.02	−2.88/0	−1.75/0
Monocarboxylate transporter 4 (SLC16A3)	O15427	49	8%	−3.34/0.03	−3.34/0.03	−3.34/0.03
CD59 glycoprotein (CD59)	P13987	14	20%	−1.83/0.03	−2.14/0.01	−3/0
Mesoderm-specific transcript homolog protein (MEST)	Q5EB52	39	21%	−2.64/0.04	−2.64/0.04	−2.64/0.04
WASH complex subunit 7 (−)	Q2M389	136	6%	−1.83/0.02	−2.09/0.03	−2.26/0.03
PCI domain-containing protein 2 (PCID2)	Q5JVF3	46	10%	−1.6/0.03	−1.32/0.02	−2.46/0.01
Cystathionine beta-synthase (CBS)	P35520	61	8%	−1.85/0.01	−1.85/0.01	−1.85/0.01
Argininosuccinate synthase (ASS1)	P00966	47	59%	6.09/0.01	3.73/0.01	4.72/0.02
UDP-glucose 6-dehydrogenase (UGDH)	O60701	55	54%	3.1/0.01	1.61/0.03	1.44/0.03
Glucosamine--fructose-6-phosphate aminotransferase [isomerizing] 1 (GFPT1)	Q06210	79	33%	3.84/0.03	2.57/0	3.58/0.01
Annexin A4 (ANXA4)	P09525	36	48%	2.96/0.01	2/0.01	1.93/0.04
Enoyl-CoA delta isomerase 1, mitochondrial (ECI1)	P42126	33	38%	1.77/0	1.04/0.01	1.21/0.04
Tryptophan--tRNA ligase, cytoplasmic (WARS)	P23381	53	44%	1.39/0.02	2.09/0	2.03/0.01
6-phosphofructokinase, muscle type (PFKM)	P08237	85	36%	1.66/0	1.42/0.01	2.01/0.04
Dihydropyrimidinase-related protein 2 (DPYSL2)	Q16555	62	49%	4.39/0	4.18/0.03	4.49/0
Aldehyde dehydrogenase, mitochondrial (ALDH2)	P05091	56	44%	4.32/0.04	3.01/0.01	4.62/0.01
Mitochondrial inner membrane protein (IMMT)	Q16891	84	24%	1.09/0.04	1.43/0.01	1.31/0.05
Hydroxymethylglutaryl-CoA synthase, cytoplasmic (HMGCS1)	Q01581	57	35%	4.09/0.01	2.05/0	4.46/0.02
Aconitate hydratase, mitochondrial (ACO2)	Q99798	85	28%	1.21/0.03	1.14/0.04	2.19/0
Ribosome maturation protein SBDS (SBDS)	Q9Y3A5	29	51%	2.74/0.03	3.12/0	2.01/0.04
UTP--glucose-1-phosphate uridylyltransferase (UGP2)	Q16851	57	28%	3.71/0	3.37/0.02	3.8/0.02
Farnesyl pyrophosphate synthase (FDPS)	P14324	48	24%	1.63/0.03	1.42/0.03	1.81/0.01
Glyoxylate reductase (GRHPR)	Q9UBQ7	36	42%	1.7/0.05	2.52/0.02	1.84/0.03
Prolyl endopeptidase (PREP)	P48147	81	19%	3.78/0.04	2.37/0.01	2.32/0.03
Phosphoglycolate phosphatase (PGP)	A6NDG6	34	48%	3.41/0.01	3.21/0.04	2.39/0.02
Mitochondrial import inner membrane translocase subunit (TIMM44)	O43615	51	20%	3.52/0.03	4.13/0.03	1.91/0.05
Ribosyldihydronicotinamide dehydrogenase (NQO2)	P16083	26	51%	2.69/0.02	3.24/0.03	2.84/0.02
Isochorismatase domain-containing protein 1 (ISOC1)	Q96CN7	32	33%	1.86/0.03	1.81/0.02	2.85/0
Atlastin-3 (ATL3)	Q6DD88	61	27%	2.53/0	3.29/0.04	3.16/0.03

a The sequence coverage (cov.) is the max value across all analyses.

b p values of Student's T test are calculated based on the quantitative values from triplicate analyses. Blank values are substituted by 1, so that p values and fold changes can be calculated.

c The log2 transformed fold change (FC) is calculated by dividing the protein quantitative value of GC cell by that of GES-1. The quantitative value is the normalized weighted spectra count calculated by Scaffold 4.0.5.

Two calponin homology domains, 20 ~ 24 filamin-type immunoglobulin domains, 1 or 2 fibronection type 3 domains, and a PDB domain have been predicted in the filamin C protein using SMART program (Supplementary Fig. 2A). The sequence coverage of filamin C was 34–44% in GES-1 cells and 1.9–3.5% in SGC-7901 and MGC-803 cells. Comparing with filamin C isoform b, filamin C isoform a has an additional 33 amino acids, which is generated by alternative splicing, between sites 1,734 and 1,766. A tryptic peptide covering this fragment of 33 amino acids and its adjacent regions was identified in the GES-1 cell line with three replicates of LC-MS analysis, suggesting that the isoform a of filamin C was detected in the present study (Supplementary Fig. 2A). One of five spectra of this peptide was shown in Supplementary Fig. 2B.

### Filamin C is downregulated in GC cell lines and GC tissues

**Figure 2:**
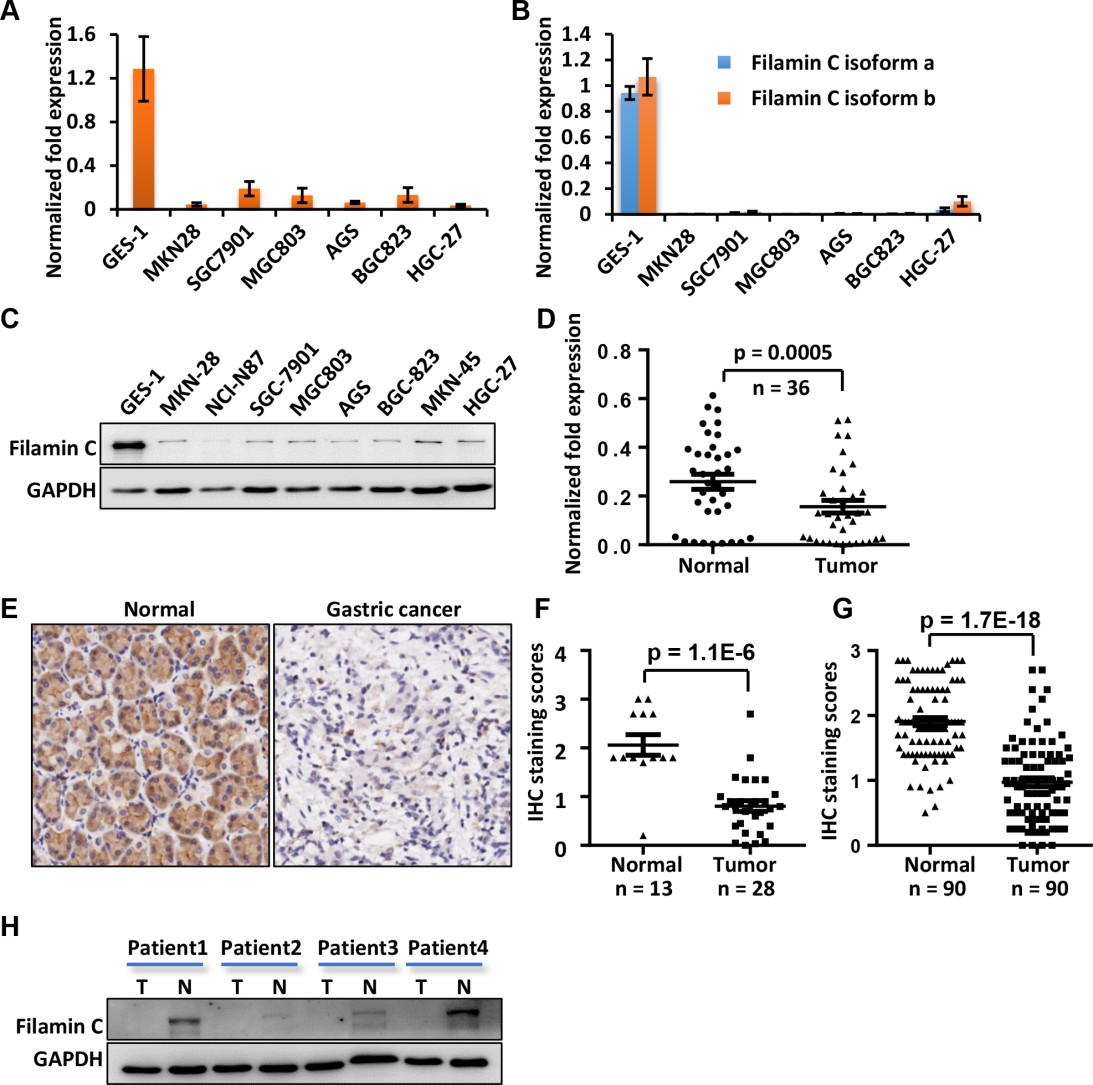
The mRNA and protein levels of filamin C were significantly reduced in GC cell lines and GC tissues compared with the gastric cell line and corresponding normal gastric tissues **(A)** The *filamin C* mRNA in GC cell lines was measured using qPCR. **(B)** The mRNA of both isoforms a and b of *filamin C* were detected in GC cell lines. **(C)** The filamin C protein levels in GC cell lines were analyzed by Western blot. **(D)** The mRNA of *filamin C* was measured by qPCR in 36 pairs of human GC and the normal gastric tissues. **(E)** Filamin C expression in GC tissues was analyzed using a commercial TMA (Shanghai Outdo Biotech, China). **(F)** Statistical analysis of the expression of filamin C in 28 GC tissues and 13 normal gastric tissues included in a commercial TMA sample collection. **(G)** Statistical analysis of the expression of filamin C in 90 GC tissues and 90 normal gastric tissues included in another commercial TMA sample collection. **(H)** A Western blot analysis of filamin C expression in four pairs of GC samples and normal gastric tissues for which the mRNA levels were determined using qPCR as shown in Figure 2D.

To verify the results of proteomic analyses, we compared the expression of filamin C in GES-1 and 6 GC cell lines including the three used in the LC-MS analysis using quantitative reverse transcription (qPCR) and Western blot. Based on qPCR assay, the mRNA of *filamin C* was dramatically down-regulated in all six GC cell lines (Figure 2A). In addition, the results of qPCR with isoform-specific primers (Supplementary Table 1, Supplementary Fig. 1) demonstrated that both isoforms of *filamin C* were significantly down-regulated in all GC cell lines (Figure B). In addition, the protein level of filamin C in the 6 GC cell lines was significantly lower than that in GES-1 cells based on Western blot analysis, which was consistent with the qPCR results (Figure 2C).

In addition, the expression of *filamin C* was significantly reduced in 36 GC tissues compared with their corresponding normal gastric tissues based on qPCR assay (*p* = 0.002, n = 36) (Figure 4D). The expression of filamin C was also evaluated in human GC tissues from two commercial tissue microarrays (TMAs) containing different sets of GC cases. Consistently, filamin C expression was obviously reduced in these GC tissues (*p* = 1.1E-6) compared with normal gastric tissues (Figure 2E and F). In the analysis of another TMA containing 90 pairs GC and normal counterpart tissues, filamin C was significantly downregulated in GCs (*p* = 1.7–18, paired Student's *t* test) (Figure 2G). The sample information and scoring results for the staining were deposited in Supplementary Table 5. We also analyzed the protein expression of filamin C in GC tissues using Western blot. Filamin C was low in all four GC tissues comparing with the normal counterparts (Figure 2H).

### *Filamin C* expression is significantly reduced in a variety of human cancers

**Figure 3:**
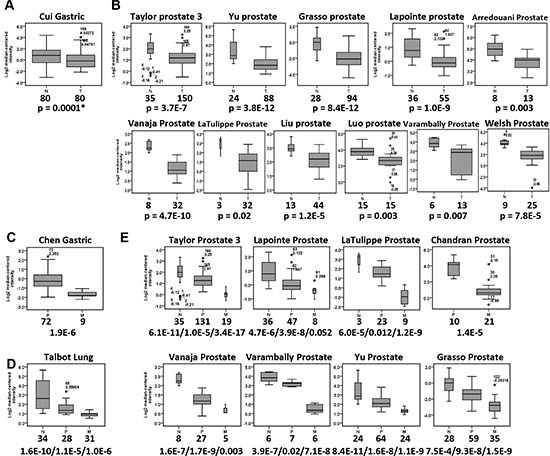
Reduced mRNA level of *filamin C* was commonly detected in various types of human cancers by analyzing the microarray datasets in Oncomine (https://www.oncomine.org/) **(A)** The *filamin C* mRNA level was significantly reduced in GC tissues based on the Cui dataset. **(B)** The *filamin C* mRNA level was significantly reduced in prostate cancer based on 11 microarray datasets. **(C)** The *filamin C* mRNA level was significantly reduced in metastatic tumors of gastric cancer based on the Chen dataset. **(D)** The *filamin C* mRNA level was significantly reduced in the lung metastatic tumors of squamous cell carcinomas (Talbot Lung). **(E)** The *filamin C* level was significantly reduced in metastatic tumor of prostate cancer compared with the normal tissues and primary prostate cancer tissues based on 8 microarray datasets. The line inside the boxes represents the median value. The box length indicates the interquartile range. The asterisk (*) indicates the extreme value > 3 interquartile ranges from the end of the box. The outliers (o) have values > 1.5 interquartile ranges but < 3 interquartile ranges from the end of the box. The boxplot and error bar width are scaled based on count. N, normal tissues; P, primary cancers; M, metastatic tissues. The numbers under the tissue type indicate the total cases of each cancer type. Statistical significances were calculated using Student's *t* tests and a *p* value < 0.05 was considered as statistically significant. The *p* values separated with slashes indicated the comparisons of normal/primary, normal/metastatic and primary/metastatic cancers.

Based on proteomics, qPCR, Western blot, and immunohistochemistry (IHC) analyses, we confirmed that the expression of filamin C was significantly reduced in GC cell lines and tissues compared with that in normal gastric cell line and tissues. Whether downregulation of filamin C is common in other human cancers is not clear. To address this question, we performed a comprehensive analysis of the expression of filamin C in other tumor tissues based on the publically available Oncomine microarray datasets (https://www.oncomine.org/). First, significantly reduced mRNA level of *filamin C* in GC tissues was identified in the Cui gastric microarray dataset (Figure 3A). Reduced expression of *filamin C* was also observed in 11 independent datasets of 561 cases of prostate cancer (Figure 3B). In addition, significantly reduced expression of *filamin C* was detected in 5 datasets of colon cancers (510 cases), 3 datasets of breast cancer (2,564 cases), 3 datasets of bladder cancer (282 cases), 4 datasets of head-neck cancers (95 cases), 1 dataset of leukemia (2,022 cases), 3 datasets of ovarian cancer (674 cases), 1 dataset of esophagus cancer (53 cases), and 4 datasets of lung cancer (410 cases) (Supplementary Figure 3). These observations suggest that *filamin C* is downregulated in a broad range of human cancers.

In addition, analyses of the Oncomine datasets revealed that the expression of *filamin C* was significantly reduced in the metastatic tissues compared with the primary GC tissues (*p* = 1.9E-6) (Figure 3C). Significantly reduced expression of *filamin C* was also found in the primary squamous cell carcinomas and the metastatic tumors in the lung compared with the normal counterparts (Figure 3D). Furthermore, the gradually decreased *filamin C* expression in normal prostate tissues, primary and metastatic prostate cancer was identified based on analyses of eight independent microarray datasets consisting of 140 normal prostate tissues, 368 primary prostate cancer and 127 metastatic prostate tumors (Figure 3E). These findings strongly suggest that reduced expression of *filamin C* is associate with the initiation and metastasis of gastric cancer, prostate cancer, and squamous cell carcinomas.

### Filamin C inhibited the proliferation of cancer cells

**Figure 4:**
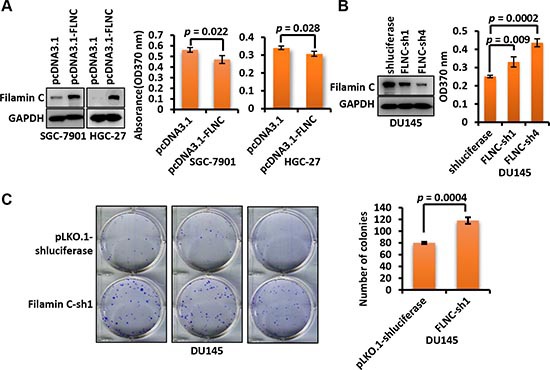
Filamin C inhibited the proliferation of GC cells **(A)** Ectopic expression of *filamin C* in SGC-7901 and HGC-27 cells cells inhibited the proliferation of cancer cells based on BrdU assay after two days of culturing. The filamin C overexpression was confirmed using Western blot (left). **(B)** Knockdown of *filamin C* by two shRNAs (sh1 and sh4) improved the proliferation of cancer cell line DU145. Filamin C knockdown was confirmed using Western blot (left). **(C)** Clone formation assay of the proliferation of DU145 cells in which endogenous *filamin C* was silenced by shRNA. The statistical analysis results of the clone numbers of DU145 cells were shown on the right. Statistical significances were calculated using Student's *t* tests and a *p* value < 0.05 was considered as statistically significant. FLNC, filamin C.

As shown in Figure 4A, ectopic expression of *filamin C* significantly inhibited the proliferation of GC cell lines SGC-7901 (*p* = 0.022) and HGC-27 (*p* = 0.028) by BrdU assay. In addition, silencing of endogenous *filamin C* using short hairpin RNAs (shRNAs) significantly improved the proliferation of prostate cancer cell line DU145 that expresses relatively high level of filamin C (*p* = 0.009 for sh1 and *p* = 0.0002 for sh4) (Figure 4B). Colony formation assay was also conducted to evaluate the proliferation of DU145 cells. The colony formation ability of DU145 cells with *filamin C* knockdown was greatly enhanced compared with the control cells (*p* = 0.0004) (Figure 4C). These experiments suggested that filamin C could inhibit cancer cell proliferation.

### *Filamin C* modulates cancer cell migration and invasion

**Figure 5:**
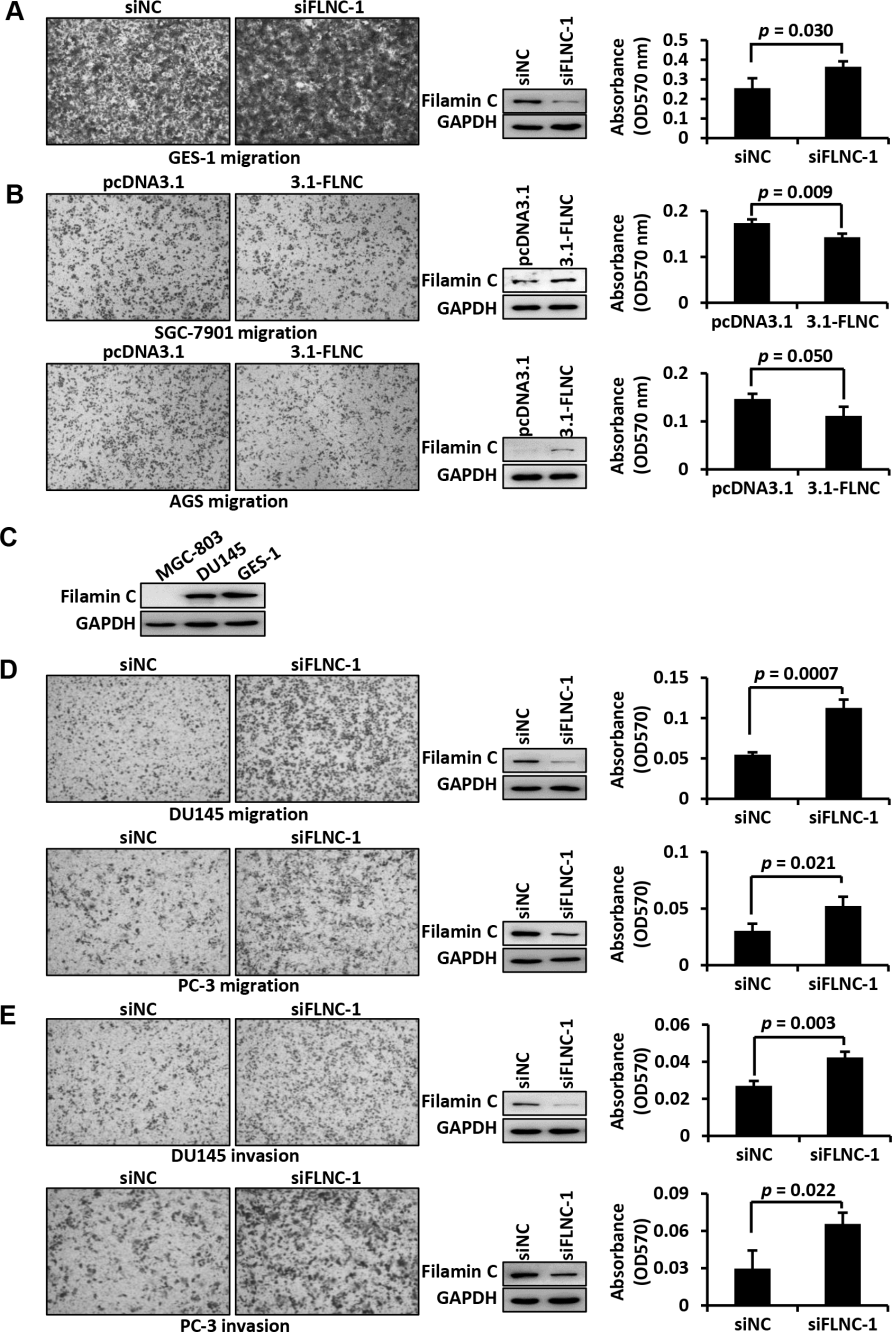
Filamin C inhibited the migration and invasion of cancer cells **(A)** *Filamin C* silencing was performed in GES-1 cells, which express relatively high level of endogenous *filamin C*. Cell migration ability was analyzed in Transwell plates and representative images of the migration cells were shown. Western blots in the middle panel showed the results of *filamin C* silencing. The cells that migrated to the bottom surface of the membrane were stained by crystal violet, which was solubilized with acetic acid and absorbance was measured under 570 nm (right panel). siNC, negative control of silencing. **(B)** *Filamin C* gene was ectopically expressed in GC cell lines and cell migration ability was analyzed in a Transwell plate. Overexpression of *filamin C* was shown in the middle panel. Cell migration was measured using the absorbance method. Statistical analysis was performed using Student's *t* test and *p* < 0.05 was considered as statistically significant. 3.1, pcDNA3.1 vector; 3.1-filamin C, expression vector pcDNA3.1-filamin C. **(C)** Western blot analysis indicated that filamin C expression in the prostate cancer cell line DU145 was similar to that in GC cell line GES-1. *Filamin C* silencing in the prostate cancer cell lines DU145 and PC-3 improved cell migration **(D)** and invasion **(E)**. Consistent results were obtained from triplicate experiments. FLNC, filamin C.

Silencing endogenous *filamin C* by siRNA in GES-1 cells significantly improved the the migration ability of GES-1 cells (*p* = 0.03) (Figure 5A). On the contrary, re-expression of *filamin C* significantly inhibited the migration of GC cell lines SGC-7901 (*p* = 0.01), AGS (*p* = 0.011), and MGC-803 (*p* = 0.021) (Figure 5B). As mentioned above, the prostate cancer cell line DU145 has a relatively high level of filamin C expression, which is comparable to that in the normal gastric cells GES-1 (Figure 5C). Silencing of endogenous *filamin C* in DU145 cells significantly improved the migration of DU145 cells (*p* = 0.0007) (Figure 5D). Similarly, partial silencing of *filamin C* considerably increased the migration of prostate cancer cell line PC-3 (*p* = 0.021) (Figure 5D). In addition, Matrigel invasion assays demonstrated that *filamin C* knockdown enhanced the invasion of DU145 (*p* = 0.003) and PC-3 cells (*p* = 0.022) (Figure 5E).

**Figure 6:**
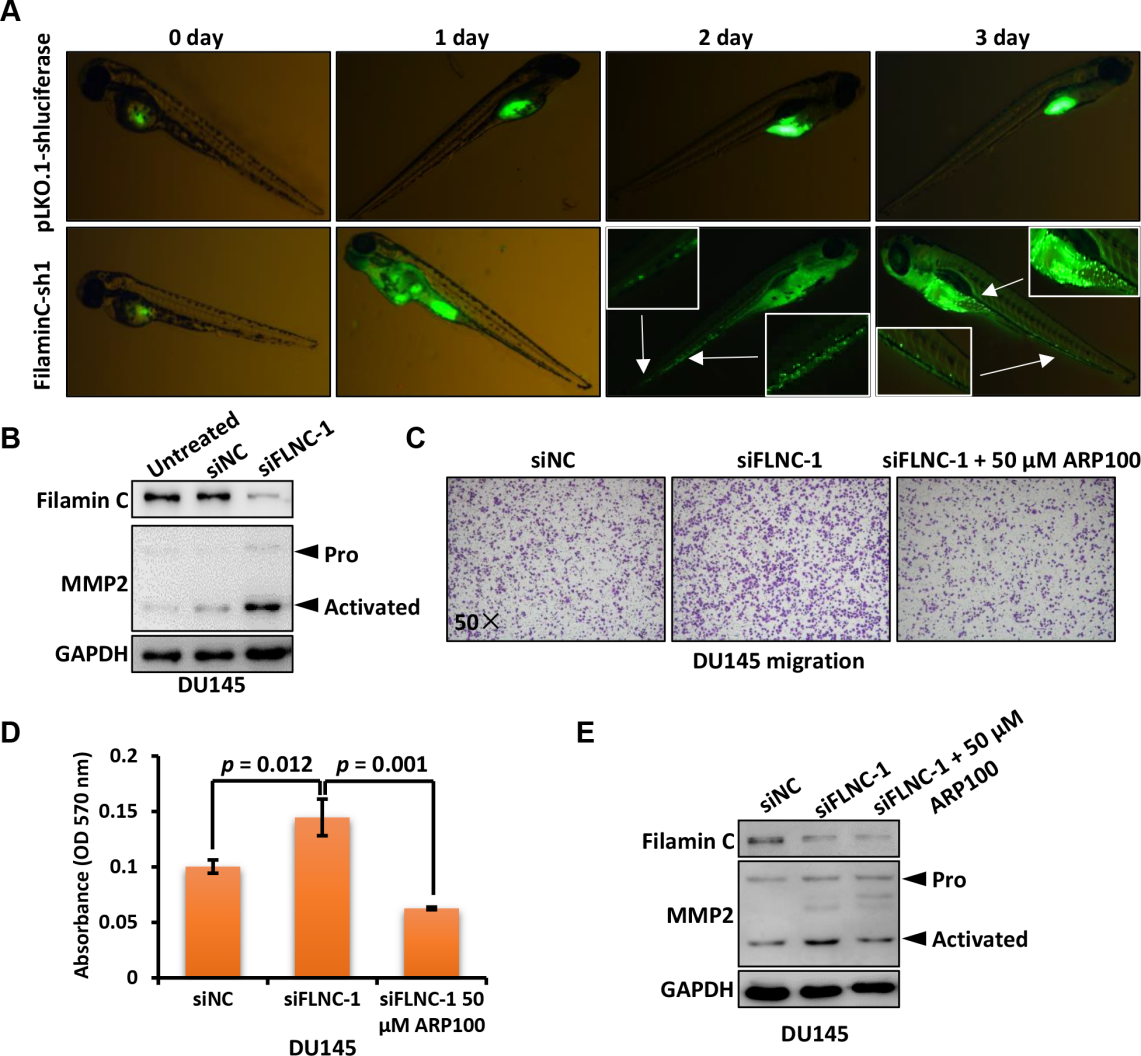
Filamin C inhibited the metastasis of cancer cells through downregulating MMP2 **(A)** DU145 cells with *filamin C* silencing were fluorescence-labeled and analyzed in a zebrafish cancer metastasis model. The selected pictures were shown from 0 to 3 days post injection. The areas indicated with arrows were enlarged for visualizing disseminated cancer cells. **(B)** *Filamin C* was silenced in DU145 cells and the proenzyme (Pro) and activated form of MMP2 were analyzed. **(C)** Migration of DU145 cells with or without *filamin C* silencing were analyzed. The third group with filamin C silencing was further treated with the MMP2 inhibitor ARP100. The statistical analysis results were shown in Figure 6D and the Western blot results were shown in Figure 6E. FLNC, filamin C.

We then used a zebrafish cancer metastasis model to investigate the role of filamin C in metastasis of cancer cells *in vivo*. Fluorescence dye-labeled DU145 cells were inoculated into the yolk of 48 hpf zebrafish and cell dissemination was measured in the following 3 days. As shown in Figure 6A, DU145 cells with stable *filamin C* silencing disseminated in the fish york after 24 h post injection and continued to spread to the head and tail of fish compared with the fish inoculated with DU145 cells without *filamin C* silencing. Collectively, *filamin C*-sh1 group had 36 (73.5%) metastasis-positive and 13 (26.5%) metastasis-negative fish, while pLKO.1-shluciferase group had less metastasis-positive (n = 25, 45.5%) but more metastasis-negative (*n* = 30, 54.4%) fish (Table 2). Significant difference was identified between the two groups based on Chi-square test (*p* = 0.004).

**Table 2:** Development of micrometastases after xenotransplantation of DU145 cells in the zebrafish model

Category	pLKO.1-shluciferase	Filamin C-sh1	X^2^	*p* value
Total number of embryos with tumors	55	49		
Dissemination in yolk	10 (18.2%)	22 (44.9%)		
Dissemination in tail	15 (27.3%)	12 (24.5%)		
Dissemination in head	0	2 (4.1%)		
Total dissemination	25 (45.5%)	36 (73.5%)	8.386	0.004
No dissemination	30 (54.5%)	13 (26.5%)		

Matrix metallopeptidases (MMPs) were known to be involved in the degradation of extracellular matrix and cancer metastasis [24]. We found that downregulated *filamin C* induced the upregulation of active MMP2 in DU145 cells (Figure 6B). Then, we performed a Transwell migration assay of DU145 cells with or without *filamin C* silencing. Furthermore, one set of tested cells with *filamin C* silencing were treated with 50 μM MMP2 inhibitor ARP100 (Figure 6C). The results showed that *filamin C* knockdown significantly increased the cell migration rate, whereas ARP100 treatment reversed this effect (Figure 6D). Western blot result revealed that MMP2 expression was upregulated upon filamin C silencing but reduced by ARP100 treatment (Figure 6E). These results suggest that filamin C inhibited the metastasis of cancer cells, may through downregulating MMP2.

### Association of filamin C expression with survival

To address the relationship of filamin C with the prognosis of GC patients, we performed an IHC analysis using another commercial TMA containing 90 pairs of GC specimens. A Kaplan-Meier survival analysis indicated that the protein level of filamin C was not significantly associated with the survival of GC patients (data not shown). We then analyzed the potential prognosis significance of mRNA expression of *filamin C* using gene expression datasets from Oncomine database. We failed to find significant association of *filamin C* with the outcome of GC patients (data not shown). However, high expression of *filamin C* was found to be associated with better prognosis of prostate cancer patients (Log-rank test, *p* = 0.0024) (Supplementary Figure 4A). We also revealed that high *filamin C* could predict better outcome of breast cancer (Log-rank test, *p* = 0.0031) and leukemia patients (Log-rank test, *p* = 0.044) (Supplementary Figure 4B and C).

## DISCUSSION

In this study, we identified 2,750 proteins by comparing the proteomic profiles of three GC cell lines and a normal gastric cell line. Nine proteins were significantly downregulated in the three GC cell lines compared to the normal gastric cell line. Among the nine proteins, UCHL1 is a member of the family of deubiquitinating enzymes and functions as a tumor suppressor in hepatocellular carcinoma and a high methylation (77%) of the gene encoding UCHL1 was found in primary GCs [25]. However, there is evidence suggests that UCHL1 is an oncogene in some cancers, such as colon cancer [26]. Transgelin is also a tumor suppressor in several types of cancers [27]. PYGL is a liver-type of glycogen phosphorylase and mutation of its gene is associated with high risk of relapse of childhood acute lymphoblastic leukemia [28]. EPHA2, UAP1, HEATR2, MBOAT7, and NOP16 have relative small quantitative values based on label-free MS quantification. As a receptor tyrosine kinase overexpressed in a variety of human cancers, EPHA2 has been extensively studied and represents a novel drug target for cancer therapeutics [29]. The roles of UAP1, HEATR2, and MBOAT7 in cancer remains unclear. NOP16, or HBV pre-S2 trans-regulated protein 3, is revealed to be an estrogen and c-Myc target gene which is upregulated in breast cancer and associated with poor prognosis of breast cancer patients [30].

Our analysis revealed that filamin C was the most differentially expressed protein in GC cell lines compared with the protein level in normal gastric cells. Filamin C belongs to the filamin family. This protein is a muscle-specific filamin and a large actin-cross-linking protein. Reorganization of filamentous cytoskeleton components was associated with many cellular processes including oncogenic transformation [31]. Filamin family includes isoforms A, B and C, which are multidomain and actin-binding proteins involved in the organization of actin cytoskeleton and linking cell surface adhesion proteins [32]. Interestingly, one of the filamin members, FLNA, is reported to suppress cancer cell migration and invasion and modulate focal adhesion [33]. It has been reported that mutations in the actin-binding domains and immunoglobulin-like domains of filamin C are associated with muscle diseases [34]. The previous proteomic study by Deng *et al*. [35] failed to identify filamin C in GES-1 cells, which may be caused by the fact that the iTRAQ method used in this study was not able to detect proteins with significantly high fold changes.

It has been shown that the *filamin C* promoter was frequently methylated in several types of human cancers. In 2002, Kaneda *et al*. reported that a CpG island of the *filamin C* promoter was methylated in 66.7% of GC cell lines including MKN28 cell line and 41.5% of primary GC tissues [36]. The reported methylation frequencies of the *filamin C* promoter in GC tissues in other studies were 44 of 119 (37%) [37] or 31/75 (41.3%) [38] GC tissues. *Filamin C* promoter methylation is considered to be induced by *H. pylori* infection and the methylation rate was reduced with eradication of *H. pylori* infection [39]. In addition, methylation of the *filamin C* promoter is also found in prostate cancer [40, 41], ovarian cancer [42, 43], and colon cancer [44].

The methylation in the promoter region of *filamin C* is expected to impair its transcriptional activity, which has been documented in GC [38]. In the present study, we provided evidence showing that filamin C was significantly downregulated at the mRNA and protein levels in GC cell lines, including MKN28 in which *filamin C* promoter methylation has been reported (Figure 3B) [36]. Furthermore, our qPCR results demonstrated that *filamin C* mRNA was considerably downregulated in GC tissues compared with corresponding normal gastric mucosa tissues. IHC analyses using two different sets of TMAs also showed that the filamin C protein level was significantly reduced in GC tissues compared with the normal counterparts. Western blot analysis of four pairs of GC and normal tissues also revealed the downregulation of filamin C in GC tissues. In addition, the mRNA level of *filamin C* was significantly reduced in a variety of human cancers. Based on these results, we hypothesize that filamin C is a potential tumor suppressor that is involved in the initiation and development of GC and other human cancers. The higher level of filamin C promoter methylation has been associated with higher risk of GC [45] and poor survival of GC patients [37]. Similarly, DNA methylation of *filamin C* promoter in colon cancer was associated with poor survival of the patients [44]. The CpG island of *filamin C* gene is also found to be hypermethylated in prostate cancer and associated with the systematic relapse of prostate cancer [40, 41].

Despite the above findings, the biological function of filamin C in cancer is not fully understood. Previous study has suggested that filamin C was not involved in carcinogenesis [42]. To answer this question, we performed gene silencing and overexpression analyses of filamin C in GC and prostate cancer cell lines. We found ectopic expression of filamin C inhibited the proliferation of the GC cell lines SGC-7901 and HGC-27. While silencing of endogenous *filamin C* improved cancer cell proliferation and colony formation. These results suggest filamin C might be involved in the proliferation of cancer cell.

Finally, our results and findings from other studies suggest that *filamin C* plays a role in cancer metastasis. Kim *et al.* revealed that promoter methylation of *filamin C* was more common in metastatic tumors of GC than in the primary GC [46]. Reduced expression of filamin C has also been observed in the metastatic tumor of prostate cancer [47]. Furthermore, filamin C mRNA is reduced in the metastatic tissues of squamous cell carcinoma and prostate cancer (Figure 3B and 3C). In the present study, silencing of endogenous *filamin C* gene in the normal gastric cell line GES-1 significantly increased the migration of GES-1 cells, which is consistent with the previous studies mentioned above. In addition, ectopic expression of full length *filamin C* gene in GC cell lines SGC-7901, AGC, and MGC-803 considerably inhibited the migration of these cells. Furthermore, silencing of *filamin C* significantly increased the migration and invasion of prostate cancer cells. An *in vivo* analysis using zebrafish cancer metastasis model demonstrated that reduced filamin C in cancer cells increased cell metastasis. We also revealed filamin C downregulation was associated with upregulation of MMP2. These results support that *filamin C* is a tumor suppressor inhibiting the metastasis of cancer cells.

Taken together, by proteomic analysis of GC cell lines, we identified and characterized a potential tumor suppressor filamin C that was significantly downregulated in GC cell lines and tissues, as well as several other human cancers. We provided evidence showing that filamin C inhibited the proliferation and the metastasis of tumor cells. Filamin C may be a target for the development of novel anticancer drugs for the treatment of GC and other human cancers.

## MATERIALS AND METHODS

### Cell lines and cell culture

The GC cell lines NCI-N87, HGC-27, BGC-823 and MGC-803 are purchased from the Cell Bank of Shanghai Institutes for Biological Sciences, China. The cell lines SGC-7901, MKN45 and MKN28 are gifts from Dr. Jian-Jun Du at Huashan Hospital, Fudan University. GES-1 and AGS are generously presented by Dr. Qing-Hua Zhang at the Shanghai University of Traditional Chinese Medicine. Prostate cancer cell lines DU145 and PC-3 were gifts from the Cell Resource Center at the Institutes of Biomedical Sciences (IBS), Fudan University. These cells were maintained in RPM1640 media (HyClone Laboratories, China) supplemented with 10% fetal bovine serum and double antibiotics (penicillin and streptomycin) in a humidified incubator at 37°C and 5% CO_2_.

### Antibodies

Rabbit polyclonal filamin C antibody was purchased from Sigma-Aldrich (Shanghai, China). GAPDH antibody was bought from Kangwei Century Co. LTD, Beijing, China. Antibody for human MMP2 was purchased from Proteintech (Wuhan, China).

### Clinic samples used for qPCR analyses

Thirty six paired gastric carcinoma and normal mucosa biopsies were used for qPCR analyses. The specimens were collected in the First Affiliated Hospital of Soochow University, Jiangsu, China, and signed informed consent was obtained from all patients. The study was approved by the Clinical Research Ethics Committee of First Affiliated Hospital of Soochow University. These samples consisted of 33 gastric adenocarcinomas, 1 adenocarcinoma/signet ring carcinoma, 1 cancerous cancer, and 1 gastric neuroendocrine carcinoma.

### Protein quantitation and in-gel tryptic digestion

The protein concentration was determined using a BCA protein assay kit (Beyotime Inc. Haimen, China). Cellular proteins were separated in a 10% SDS-PAGE. For proteomic analyses, 40 μg proteins were loaded into each lane and an equal amount of protein sample was run in triplicate in the same gel. Each gel lane was diced into 10 slices and in-gel tryptic digestion of proteins was conducted as previously reported [48].

### Reverse phase LC-MS by linear trap quadrupole (LTQ)-Obitrap

The LC-MS methods using LTQ-Obitrap have been described previously [48]. The protein samples from GES-1, HGC-27, MGC-803 and SGC-7901 were analyzed in triplicates using LTQ Obitrap. Briefly, peptide samples were loaded one-by-one onto a CAPTRAP column (0.5 × 2 mm, Michrom Bioresources, Auburn, CA) using a SIL-20 AC auto-sampler (ThermoFisher, San Jose, CA) for desalting and concentration. A LC-20AB micro-flow LC pump (Shimadzu, Tokyo, Japan) was used for sample loading at a flow rate of 20 μL/min. The mobile phases A and B were 2% ACN/0.1% FA and 95% ACN/0.1% FA, respectively. The desalted peptides were separated by an analytical C18 RP column (0.1 × 150 mm, packed with 3 μm Magic C18-AQ particles, Michrom Bioresources, Auburn CA) at a flow rate of 500 nL/min delivered by a nano-flow LC pump LC-20AD (Shimadzu, Tokyo, Japan). A 90-min linear gradient from 2 to 45% phase B was applied. The separated peptides were ionized and sprayed via an Advance silica tip (30 μm inner diameter, Michrom Bioresources, Auburn, CA) that was connected to the end of the analytical column. The spray voltage was 1.6 kV and the heated capillary was set at 200°C. The ions were analyzed using a LTQ-Orbitrap mass spectrometer (ThermoFisher, San Jose, CA) in data-dependent mode. Each duty cycle started with one full MS survey scan at the mass range 350–1,800 Da using the Orbitrap section. The top fifteen most intense ions above 200 counts in survey scan with charges 2+ or 3+ were chosen for MS/MS fragmentation in the LTQ section using collision-induced dissociation. The mass range for each MS/MS was 220–2,000 Da. The normalized collision energy value was 35% and the dynamic exclusion window for peptide fragmentation was 90 s.

### MS Data processing and database searching

The search database contained 20, 232 human protein entries retrieved from the UniProtKB/Swiss-Prot Release 2012_07 using an in-house Perl script. Database search was performed using Mascot v2.3.2 (Matrix Science) as previously described with minor modifications [48]. Briefly, peak lists used for database search were generated from LTQ raw files using ProteoWizard v3.0 with default settings (http://proteowizard.sourceforge.net/). Mascot Daemon (Matrix Science, v2.3.2) was used to automatically search the database with the same searching parameters previously used except that peptide charges 2+ and 3+ were considered.

The database search results were parsed and imported into Scaffold v4.0.5 (Proteome Software, Inc.) for data integration and validation. An additional database search by X! Tandem (The GPM http://thegpm.org, version CYCLONE 2010.12.01.1) was performed with parameters similar to Mascot. Peptide identifications by X! Tandem were accepted with > 95.0% probability assigned using the PeptideProphet algorithm [49]. Probabilities of Mascot identifications were assigned by the Scaffold Local false discovery rate algorithm. Protein identifications with > 99.0% probability by the ProteinProphet algorithm [50] and ≥ 2 unique peptides were accepted. To meet the principles of parsimony, proteins were grouped according to the same set of peptides. The abundance of each protein was determined using the normalized weighted spectra count (nWSC) calculated by Scaffold. The normalization was performed by multiplying nWSC across samples, therefore, the total number of spectra was the same across all samples and categories. The differentially expressed proteins between GES-1 and the GC cell lines were identified by volcano plot analysis using geWorkbench 2.4.1 (http://www.geworkbench.org). To calculate the fold changes of proteins, the average nWSCs in the GC cell sets were divided by the average nWSC in GES-1 set and the ratios were Log^2^ transformed. The log^2^ fold change for each protein was plotted against the −log^10^ transformed T-test *p* value. In case where the expression value is absent, a minimum value of “1” was used so that the *p* value and fold change could be calculated. The *p* value threshold of geWorkbench was set at 1.0 in order to display the data points as many as possible. Proteins with consistent changes by > 2 or < 0.5 fold (Log(2) ≥ 1 or ≤ −1) at a Student's *t* test *p* < 0.01 (−log(10) ≥ 2) across all three GC cell lines were accepted as significantly differentially expressed proteins.

### Cloning of full-length filamin C

*Filamin C* gene has two variants (GenBank accession numbers: NM_001458.4 and NM_001127487.1), which encode two isoforms of filamin C, a and b, respectively. The isoform a is 33 amino acid longer than the isoform b. To clone the full length *filamin C* gene (8,175 bp) encoding the isoform a, two fragments of the coding region of *filamin C* were amplified using primer pairs FLNCfull-1-F/FLNCd15r and FLNCd15f-EcoRI-F/FLNCd24r, respectively. Total RNAs extracted from the normal gastric cell line, GES-1, was used for gene cloning. The primers FLNCd15r and FLNCd24r were designed according to Duff. R. M., *et al* [51]. The PCR amplicons were digested with restriction enzymes *EcoRI* and *SalI* (FastDigest enzyme, ThermoFisher Scientific, Shanghai, China) and inserted into plasmid pBABE-puro. The two fragments were then cleaved from the plasmid using *BglII* and ligated using T4 ligase to generate the full coding sequence (CDS) of *filamin C* gene. The full CDS was then inserted into plasmid pBABE-puro and the sequence of *filamin C* full CDS was verified by DNA sequencing. The *filamin C* gene cloned harbored 6 single-nucleotide polymorphisms (SNPs) in the CDS region compared with the reference sequence (GenBank accession NM_001458.4). For overexpression assay, *filamin C* CDS was amplified from pBABE and sub-cloned into pcDNA3.1. The primers were listed in Supplementary Table 1 and the positions of primers were shown in Supplementary Fig. 1.

### Western blot

Thirty μg cellular proteins were separated in 10% SDS-PAGE and transferred onto an Immobilon-P Transfer Membrane (Merck Millipore, Shanghai, China). Western blot assay was performed using the standard protocol with the first antibody diluted at 1:1,000. The second antibody was diluted at 1:5,000. Chemiluminescent was developed using freshly prepared SuperSignal West Femto Maximum Sensitivity Substrate (Thermo Scientific Pierce, Shanghai, China). Signal was detected using a LAS-3000 imager (Fujifilm, China).

### Cell transfection and *filamin C* knockdown via RNA interference (RNAi)

Transfection was performed using Lipofectamine 2000 or X-treme gene HP DNA transfer reagent (Roche, Shanghai, China) and OPTI-MEM reduced serum media (Life Technologies - Invitrogen, Shanghai, China) according to the manufacturers' instruction. For RNAi assay, small interference RNAs (siRNAs), including sifilamin C-1, 2, 3 and a negative control (siNC) (Supplementary Table 1), were purchased from Shanghai GenePharma Co., Ltd. For transfection, 120 pmol (GenePharma, Shanghai, China) or 100 pmol (Santa Cruz, Shanghai, China) siRNAs were used to transfect GC cell lines using Lipofectamine 2000 and opti-MEM.

### 5-Bromo-2-deoxyuridine (BrdU) assay

The proliferation of cancer cells (SGC-7901 and DU145) was evaluated using the BrdU Cell Proliferation ELISA Kit (Roche, Shanghai, China) according to the manufacturer's instruction. Cells were seeded in a 96-well plate at a density of 2,000/well and cultured for two days. The cells were labeled with 10 μM BrdU labeling reagent for 3 h, aspirated, and dried at 60°C for 1 h. The cells were then treated with FixDenat Solution for 30 min at room temperature and incubated with BrdU-POD reagent containing the antibody against BrdU. The plate was washed with washing buffer for three times. The signal was developed by adding 100 μL substrate solution containing tetramethyl-benzidine. The absorbance at OD370 nm was measured using a BioTek Epoch Microplate Spectrophotometer.

### Migration and invasion assay

GES-1, SGC-7901, AGS, DU145 and PC-3 were used for migration assay. DU145 and PC-3 were used for invasion assay. After two days of transfection, the cells were transferred to the upper chamber of a 24-well Transwell plate (Corning Life Sciences, Shanghai, China), with 0.5-1 × 10^5^ cells/well in 200 μL serum-free medium. A volume of 500 μl of 10% fetal bovine serum-containing medium was added to the lower chamber as a chemoattractant. One day after incubation at 37°C, the upper chamber was washed with 1 × PBS (pH 7.4) and treated with 4% paraformaldehyde for 10 min. Then the chamber was stained with 0.1% crystal violet for 10–15 min, followed by washing with 1 × PBS for three times to remove residue dye. Non-migrating cells on the upper membrane surface were scraped off with cotton swabs. The dye was dissolved by 33% acetic acid and the absorbance was measured at 570 nm using the spectrophotometer. Each experiment was performed in triplicate. For MMP inhibition analysis, cancer cells were transfected with *filamin C* siRNA. After cultured for 36 h, the cells transfected with *filamin C* siRNA were treated with 50 μM ARP100 MMP2 inhibitor (Apexbio, Shanghai, China) and cultured for 12 h before the evaluation of inhibitory effects. Statistical significance was analyzed using the Student's *t* test and a *p* value less than 0.05 was considered as statistically significant.

Invasion assay was performed in a similar way as the migration assay using a cell invasion assay kit (BD Biosciences, Shanghai, China) according to the manufacture's instruction, except that the upper chambers were pre-coated with ECMatrix^TM^ gel.

### Bioinformatics

GO annotation of identified proteins was retrieved via Scaffold on May 20, 2013, from National Center for Biotechnology Information (NCBI) (http://www.ncbi.nlm.nih.gov/). GO enrichment analysis was performed using DAVID functional annotation tool (http://david.abcc.ncifcrf.gov) with default settings. Venn diagram was generated using VENNY (http://bioinfogp.cnb.csic.es/tools/venny/index.html) or BioVenn (www.cmbi.ru.nl/cdd/biovenn). The protein domains of filamin C is predicted using the Simple Modular Architecture Research Tool (SMART, http://smart.embl-heidelberg.de/). *Filamin C* mRNA expression data in different types of human cancers were retrieved from the Oncomine cancer gene expression microarray database (https://www.oncomine.org). The Log^2^ median-centered intensity values of *filamin C* expression were used to generate box plots using PASW Statistics 18.

### RNA extraction, reverse transcription and qPCR

Tumor tissues were grinded in liquid nitrogen and total RNA was isolated using TRIZOL reagent (Life Technologies – Invitrogen, Shanghai, China) according to the manufactory's protocol. First strand cDNA was synthesized using the PrimeScript 1st Strand cDNA Synthesis Kit (Takara Bio Inc. Shanghai, China). qPCR was performed using the IQ5 Real-Time PCR detection system (Bio-Rad, Shanghai, China) with SYBR green (Takara Bio Inc. Shanghai, China). Primers were listed in Supplementary Table 1. Statistical significance was analysed using the Student's *t* test and a *p* value less than 0.05 was considered as statistically significant.

### Lentivirus production

The shRNA lentiviral plasmid (pLKO.1) was obtained from The RNAi Consortium. The shRNA sequences for *filamin C* were listed in Supplementary Table 1. Lentivirus was produced using HEK293FT cells with the second-generation packaging system psPAX2 (Addgene plasmid 12260) and pMD2.G (Addgene plasmid 12259). Lentiviral titer was determined by measuring viral RNA content in viral supernatant using qPCR as previously described [52].

### Colony formation assay

DU145 cells (lentivirus-transfected strain) were seeded in a six-well plate at a density of 200 cells per well and cultured at 37°C and 5% CO_2_. Two weeks latter, the culture media were aspirated and the cells were rinsed triple times with PBS before stained with 0.1% crystal violet for 10–15 min. The solution was removed and the cells then were washed triple times with PBS. The colonies were counted under microscope.

### Zebrafish cancer metastasis assay

The wild-type zebrafish (*Danio rerio*) were maintained at a density of 20 fish of both sexes per tank in an aquaria with a recirculating water system on a 12 h light/14 h dark cycle. Habitat water was buffered with non-iodized salt and sodium bicarbonate to keep the salinity at 500 μF and pH at 7.2. Fish were fed with dry brine shrimp or fish flakes according to the previous protocol [53]. Two pairs of adult fish of 3 month old (2 males and 2 females) were separately maintained in a spawning tank and allowed to mating and spawn. Juvenile fish were fed with rotifers. The next day, embryos were collected and cultured at 28°C for 2 – 4 h and the medium was changed with 1 × embryo culture medium (15 mM NaCl, 0.5 mM KCl, 1 mM CaCl_2_•2H_2_O, 0.15 mM KH_2_PO_4_, 0.05 mM NaHPO_4_, 0.1 mM MgSO_4_•7H_2_O, and 8 μM NaHCO_3_). Suspended cancer cells (3 × 10^6^ cell/mL) were stained with 5 μM Dio green fluorescent probe dye (YESEN, Ltd. Co., Shanghai, China) at 37°C for 20 min. The cells were precipitated with gentle centrifugation, washed with PBS, and suspended in 100 μL PBS for fish injection. The juvenile fish of 48 hours post fertilization (hpf) were anaesthetized with 0.016% Tricaine (Sigma-Aldrich, Shanghai, China) and placed on a apparatus made by 1.5% agarose gel, where each fish was injected into the yolk with 50–100 labeled cells in a 2-nL volume. The injected fish were maintained in 28°C for 2h and transferred to a 34°C incubator for a further incubation for 72 h. The dissemination of cancer cells in fish was pictured under a LEICA M205 FA stereo fluorescence microscope.

### Evaluation of filamin C expression in two TMAs based on IHC

Two commercial TMAs were used to analyze the expression of filamin C in GC tissues based on IHC analysis. One TMA consisted of 13 paraneoplastic tissues and 28 gastric adenocarcinoma tissues (catalog no. HStm-Ade076Met-01, Shanghai Outdo Biotech, China) and another TMA contained 90 pairs of gastric adenocarcinoma tissues and corresponding normal mucosa tissues (catalog no. HStm-Ade180Sur-04, Shanghai Outdo Biotech, China). In the second TMA, the surgical time was from May 2007 to February. 2008 with available follow-up information from May 2007 to Aug. 2013. The survival time was 1 ~ 75 months. IHC analysis was performed according to the manufacture's instruction and the IHC staining was scanned using Scanscope XT (Aperio, Shanghai, China). The expression of filamin C was scored by manually assessing the staining intensity and the frequency. Staining intensity was scored as 0 (negative), 1 (low), 2 (medium), and 3 (high). Frequency was scored as 0 (negative staining) to 95% according to the proportion of cells with positive filamin C staining. The expression of IHC was calculated by multiplying the intensity score and the frequency. The Kaplan-Meier survival curves were calculated using the Log-rank test with GraphPad Prism 6.01. *P* value < 0.05 was considered statistically significant.

## SUPPLEMENTARY FIGURES AND TABLES









## Abbreviations

2DE: two-dimensional electrophoresis
BrdU: 5-Bromo-2-deoxyuridine
DIGE: 2DE-difference gel electrophoresis
EPHA2: ephrin type-A receptor 2
GC: Gastric cancer
GO: Gene ontology
HEATR2: HEAT repeat-containing protein 2
ICAT: isotope coded affinity tag
LC-MS: liquid chromatograph-mass spectrometry
MBOAT7: lysophospholipid acyltransferase 7
MMP2: matrix metalloproteinase-2
MudPIT: multidimensional protein identification technology
NCBI: National Center for Biotechnology Information
NOP16: nucleolar protein 16
nWSC: normalized weighted spectra count
PYGL: glycogen phosphorylase
qPCR: quantitative reverse transcription-PCR
SMART: Simple Modular Architecture Research Tool
TP53: tumor protein p53
UAP1: UDP-N-acetylhexosamine pyrophosphorylase
UCHL1: carboxyl-terminal hydrolase isozyme L1
