# Supplementary material for: Filamin C, a dysregulated protein in cancer revealed by label-free quantitative proteomic analyses of human gastric cancer cells
Source: Oncotarget. 2014 Nov 28;6(2):1171–89. doi: 10.18632/oncotarget.2645 (PMC4359225; doi:10.18632/oncotarget.2645)
Supplement: Supplementary file 1 [file oncotarget-06-1171-s001.pdf]

SUPPLEMENTARY FIGURES AND TABLES

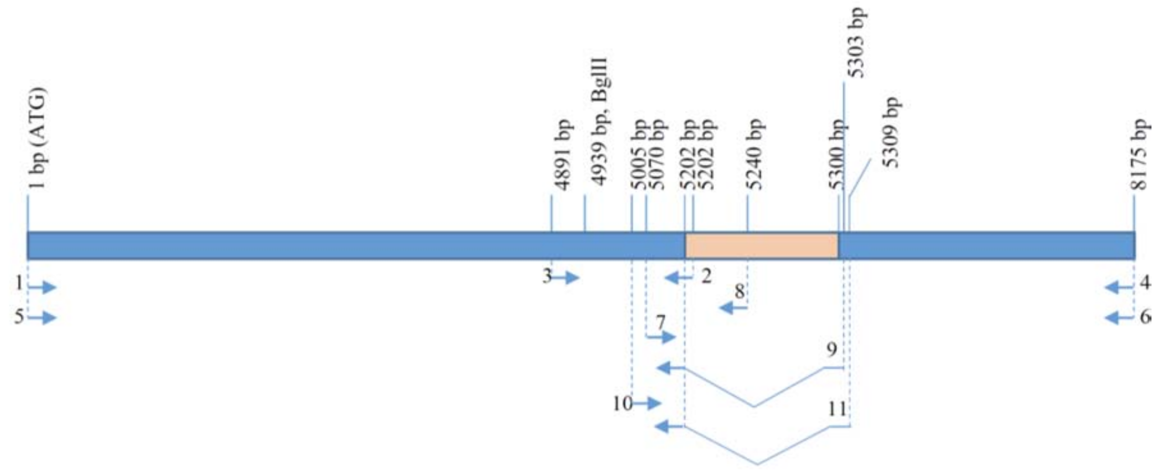

B

5' primer	Name	Position	3' primer	Name	Position	Usage
1	FLNCfull-1-F	1 bp	2	FLNCd15r	5,208 bp	5 kb fragment cloning
3	FLNCd15f-EcoRI-F	4,891 bp	4	FLNCd24r	8,175 bp	3 kb fragment cloning
5	FLNCNotI-2-F	1 bp	6	FLNCEcoRI-2-R	8,175 bp	pcDNA3.1
7	FLNC-αβ-2-F	5,070 bp	8	FLNC-α-2-R	5,240 bp	qPCR
7	FLNC-αβ-2-F	5,070 bp	9	FLNC-β-2-R	5,303 bp	qPCR
10	FLNC-β-3-F	5,005 bp	11	FLNC-β-3-R	5,309 bp	qPCR

Supplementary Figure S1: The positions of PCR or cloning primers of *filamin C*. **(A)** The positions of primers of *filamin C*. The long blue rectangle indicates the full length coding sequence of *filamin C*. The orange box indicates the alternative splicing exon for isoform a. Isoform b lacks this exon. The vertical lines indicate the primer positions along the sequence. The arrows indicate the direction of the primers. The numbers represent the primer serial numbers. **(B)** The names of the primer pairs, the primer positions and their usage. FLNC, filamin C.

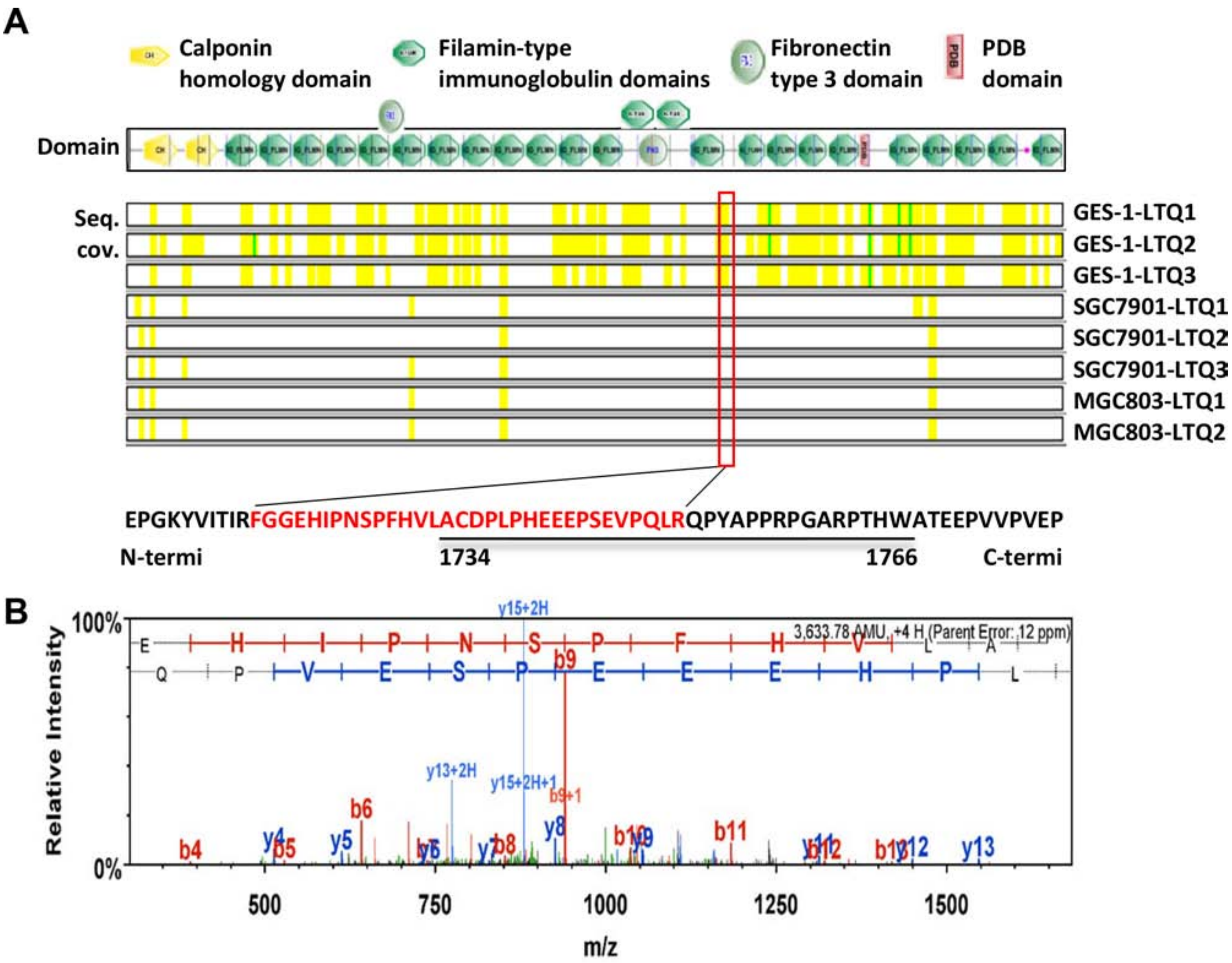

Supplementary Figure S2: *Filamin C* isoform a is identified by MS. **(A)** Filamin C is predicted by SMART to contain two calponin homology domains, 20 ~ 24 filamin-type immunoglobulin domains, 1 or 2 fibronection type 3 domains and one PDB domain. The sequence coverage (Seq. cov.) across different LC-MS analyses are shown below the domain structure. Yellow-colored boxes visually depict the fraction identified by LC-MS, whereas the green-colored positions indicate the modifications of amino acids. A red box highlights an identified peptide spanning a common region and a specific region of filamin C isoform a. The underlined sequence from amino acid 1734 to 1766 is a specific fragment of filamin C isoform a. The red peptide of this specific region is identified in GES-1. **(B)** The MSMS spectrum of the specific peptide of filamin C isoform a. The sequential b and y ions are highlighted in blue and red, respectively.

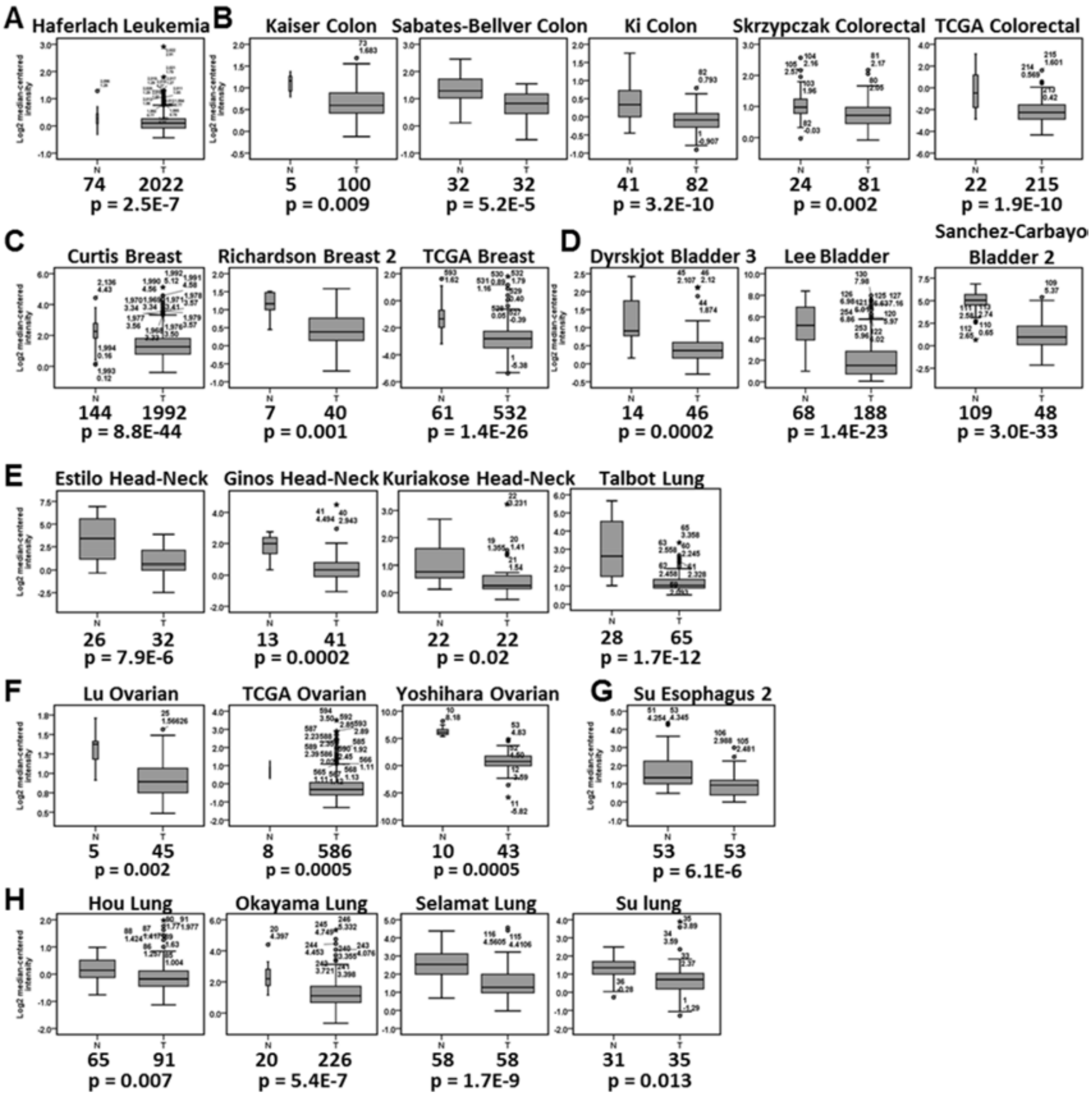

Supplementary Figure S3: *Filamin C* mRNA expression in different type of cancers were analyzed using Oncomine gene expression array database. **(A)** Leukemia dataset. **(B)** Colon cancer datasets. **(C)** Breast cancer datasets. **(D)** Bladder cancer datasets. **(E)** Head-neck cancers and the corresponding lung metastasis cancer datasets. **(F)** Ovarian cancer datasets. **(G)** Esophagus cancer dataset. **(H)**. Lung cancer datasets. The line inside the box represents the median value. The box length indicates the interquartile range (IQR). The asterisk (*) indicates the extreme value > 3 IQRs from the end of the box. The outliers (o) have values > 1.5 IQRs but < 3 IQRs from the end of the box. The boxplot and error bar width are scaled based on count. N, normal tissues; P, primary cancers; M, metastasis tissues. The numbers under the tissue type indicate the total cases for each type. Significances are calculated using Student's t tests and a p value < 0.05 was considered as statistically significant.

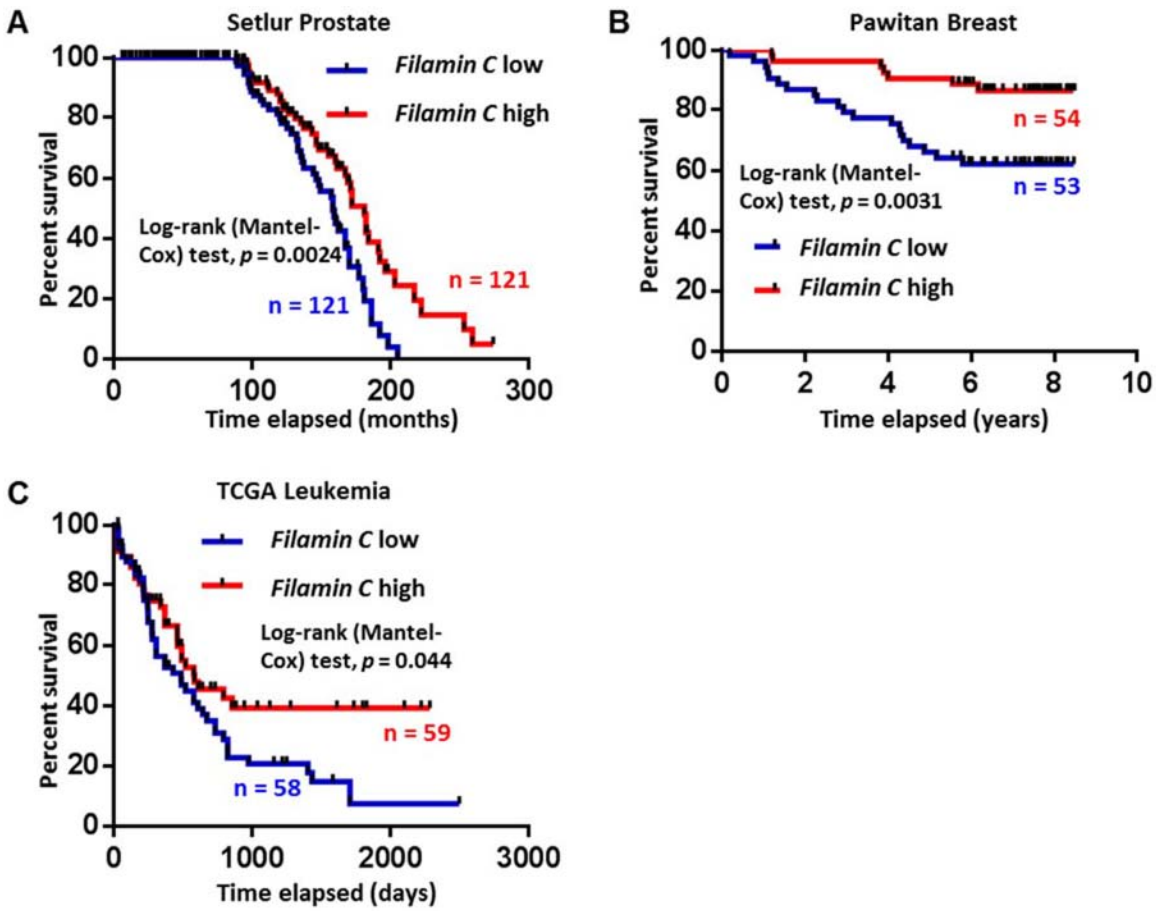

Supplementary Figure S4: Kaplan-Meier analysis of *filamin C* expression in cancer using Oncomine gene expression datasets. (A) High *filamin C* expression was significantly associated with better outcome of prostate cancer patients, as revealed by analyzing Setlur Prostate gene expression dataset in Oncomine database (www.oncomine.org). The *p* value was calculated using Log-rank (Mantel-Cox) test method. n indicates the number of cases enrolled. High expression group (*filamin C* high) was defined as the top one third of cases with higher expression of *filamin C* when all cases were ranked from the highest *filamin C* expression values to the lowest *filamin C* expression values. Low expression group (*filamin C* low) contained the bottom one third cases with lower expression of *filamin C* when all cases were ranked as above. **(B)** Survival analysis showed high *filamin C* expression predicted better prognosis in breast patients, as indicated by Pawitan Breast dataset. **(C)** Kaplan-Meier analysis of *filamin C* in leukemia patients using Oncomine dataset TCGA Leukemia.

Supplementary Table S1. Cloning primers, RT-PCR primers and siRNAs used in current study

Category Primer name	Primer sequences	Vector
**Cloning primers of *filamin C***		
FLNCfull-1-F (5' 5000 bp fragment)	CGGAATTCGCCACCATGATGAACAACAGCGGCTAC	pBABE-puro
FLNCd15r[a] (5' 5000 bp fragment)	TTTGTCGACGTCACACGCCAGCACGTGGAA	pBABE-puro
FLNCd15f-EcoRI-F (3' 3000 bp fragment)	CGGAATTCGCCACCATGCTGCCCACTGGGGATGCCAGC	pBABE-puro
FLNCd24r[a] (3' 3000 bp fragment)	TTTTGTCGACAGGGACCTTGACTTTGAAGG	pBABE-puro
FLNCNotI-2-F	GAATGCGGCCGCCACCATGATGAACAACAGCGGCTAC	pcDNA3.1
FLNCEcoRI-R	CGGAATTCAAGGGACCTTGACTTTGAAGG	pcDNA3.1
**Real-time PCR primers**		
FLNC-1-F	CAACGTGGATGAGCATTCTGT	-
FLNC-1-R	GTCCTCGATGTAGACCAGCAC	-
FLNC-α-2-R	ACTTCAGAGGGCTCCTCCTC	-
FLNC-αβ-2-F	CGATGTGGATGTGGTTGAGA	-
FLNC-β-2-R	GTGGCCAGCACGTGGAAG	-
FLNC-β-3-F	GATGCCAAGGCAGCCGGTGAG	-
FLNC-β-3-R	TCCTCTGTGGCCAGCACGTGGAAG	-
GAPDH-2-F	CGAGATCCCTCCAAAATCAA	-
GAPDH-2-R	TTCACACCCATGACGAACAT	-
**siRNAs of *filamin C***		
FLNC-homo-2954 (siFLNC-1) sense (5'-3')	GGGACUUUGAGAUCAUAGATT	-
FLNC-homo-2954 (siFLNC-1) antisense (5'-3')	UCUAUGAUCUCAAAGUCCCTT	-
FLNC-homo-6402 (siFLNC-2) sense(5'-3')	CACUCGCAAUGCAGGUUAUTT	-
FLNC-homo-6402 (siFLNC-2) antisense (5'-3')	AUAA CCUGCAUUGCGAGUGTT	-
Negative control (siNC) sense(5'-3') ;	UUCUCCGAA CGUGUCACGUTT	-
Negative control (siNC) antisense (5'-3')	ACGUGACACGUUCGGAGAATT	-
**shRNAs of *filamin C***		
FLNC-sh1-F	CCGGGCTAAGGTGGTTCCCAACAATCTCGAGATTGTTGGGAACCACCTTAGCTTTTTG	
FLNC-sh1-R	AATTCAAAAAGCTAAGGTGGTTCCCAACAATCTCGAGATTGTTGGGAACCACCTTAGC	
FLNC-sh4-F	CCGGCGGTACCTTTGACATCTACTACTCGAGTAGTAGATGTCAAAGGTACCGTTTTTG	

(*Continued*)

Category	Primer name	Primer sequences	Vector
	FLNC-sh4-R	AATTCAAAAACGGTACCTTTGACATCTACTACTCGAGTAGTAGATGTC AAAGGTACCG	
	pLKO.1-shLuciferase	GTGCGCTGCTGGTGCCAAC	

[1]The primers were designed as reported (Duff, R. M., Tay, V., Hackman, P., Ravenscroft, G., McLean, C., Kennedy, P., Steinbach, A., Schoffler, W., van der Ven, P. F., Furst, D. O., Song, J., Djinovic-Carugo, K., Penttila, S., Raheem, O., Reardon, K., Malandrini, A., Gambelli, S., Villanova, M., Nowak, K. J., Williams, D. R., Landers, J. E., Brown, R. H., Jr., Udd, B., and Laing, N. G. (2011). Mutations in the N-terminal actin-binding domain of filamin C cause a distal myopathy. American journal of human genetics 88, 729–740.)

Supplementary Table S2. The statistics of the proteomic analyses

Category	Bio Sample	#Prot[a]	#IDs[b]	#Spectra	%IDs
GES-1	GES-1-LTQ1	1132	22523	89861	0.25064266
GES-1	GES-1-LTQ2	1234	27369	98165	0.2788061
GES-1	GES-1-LTQ3	1159	20564	80216	0.25635785
SGC7901	SGC7901-LTQ1	2060	43939	126834	0.3464292
SGC7901	SGC7901-LTQ2	1310	13929	105116	0.13251075
SGC7901	SGC7901-LTQ3	1433	16807	102140	0.16454867
MGC803	MGC803-LTQ1	2086	43304	118653	0.36496338
MGC803	MGC803-LTQ2	1957	41697	110752	0.37648982
MGC803	MGC803-LTQ3	1772	35270	110535	0.31908447
HGC27	HGC27-LTQ1	1294	29245	94064	0.31090534
HGC27	HGC27-LTQ2	1315	32342	96368	0.33560932
HGC27	HGC27-LTQ3	1186	20692	72090	0.2870301
Total count			347681	1204794	

[a]Number of identified proteins, including target and decoy identifications fulfilling the cutoff.
[b]Number of spectra associated only with this protein.

Supplementary Table S3. All peptides identified from the four gastric cell lines.

Supplementary Table S4. All identified proteins from GES-1, SGC-7901, MGC-803 and HGC-27.

Supplementary Table S5. The immunohistochemistry analysis scoring of filamin C and the clinicopathological factors of the gastric cancer patients of the tissue microarray.
